# Performance analysis of pretrained convolutional neural network models for ophthalmological disease classification

**DOI:** 10.5935/0004-2749.2022-0124

**Published:** 2023-03-08

**Authors:** Busra Emir, Ertugrul Colak

**Affiliations:** 1 Department of Biostatistics, Faculty of Medicine, Izmir Katip Celebi University, Izmir, Turkey; 2 Department of Biostatistics, Faculty of Medicine, Eskisehir Osmangazi University, Eskisehir, Turkey

**Keywords:** Neural networks, computer, Deep-learning, Image processing, computer-assisted, VGG16, Inceptionv3, ResNet50, Fundus oculi, Eye diseases, Redes neurais de computação, Aprendizado profundo, Processamento de imagem assistida por computador, VGG16, Inceptionv3, ResNet50, Fundo de olho, Oftalmopatias

## Abstract

**Purpose:**

This study aimed to evaluate the classification performance of pretrained convolutional neural network models or architectures using fundus image dataset containing eight disease labels.

**Methods:**

A publicly available ocular disease intelligent recognition database has been used for the diagnosis of eight diseases. This ocular disease intelligent recognition database has a total of 10,000 fundus images from both eyes of 5,000 patients for the following eight diseases: healthy, diabetic retinopathy, glaucoma, cataract, age-related macular degeneration, hypertension, myopia, and others. Ocular disease classification performances were investigated by constructing three pretrained convolutional neural network architectures including VGG16, Inceptionv3, and ResNet50 models with adaptive moment optimizer. These models were implemented in Google Colab, which made the task straight-forward without spending hours installing the environment and supporting libraries. To evaluate the effectiveness of the models, the dataset was divided into 70%, 10%, and 20% for training, validation, and testing, respectively. For each classification, the training images were augmented to 10,000 fundus images.

**Results:**

ResNet50 achieved an accuracy of 97.1%; sensitivity, 78.5%; specificity, 98.5%; and precision, 79.7%, and had the best area under the curve and final score to classify cataract (area under the curve = 0.964, final score = 0.903). By contrast, VGG16 achieved an accuracy of 96.2%; sensitivity, 56.9%; specificity, 99.2%; precision, 84.1%; area under the curve, 0.949; and final score, 0.857.

**Conclusions:**

These results demonstrate the ability of the pretrained convolutional neural network architectures to identify ophthalmological diseases from fundus images. ResNet50 can be a good architecture to solve problems in disease detection and classification of glaucoma, cataract, hypertension, and myopia; Inceptionv3 for age-related macular degeneration, and other disease; and VGG16 for normal and diabetic retinopathy.

## Abstract (pt)

**Objetivo:**

Avaliar o desempenho de classificação de modelos ou arquiteturas de rede neural convolucional pré--treinadas usando um conjunto de dados de imagem de fundo de olho contendo oito rótulos de doenças diferentes.

**Métodos:**

Neste artigo, o conjunto de dados de reconhecimento inteligente de doenças oculares publicamente disponível foi usado para o diagnóstico de oito rótulos de doenças diferentes. O banco de dados de reconhecimento inteligente de doenças oculares tem um total de 10.000 imagens de fundo de olho de ambos os olhos de 5.000 pacientes para oito categorias que contêm rótulos saudáveis, retinopatia diabética, glaucoma, catarata, degeneração macular relacionada à idade, hipertensão, miopia, outros. Investigamos o desempenho da classificação de doenças oculares construindo três arquiteturas de rede neural convolucional pré-treinadas diferentes, incluindo os modelos VGG16, Inceptionv3 e ResNet50 com otimizador de Momento Adaptativo. Esses modelos foram implementados no Google Colab o que facilitou a tarefa sem gastar horas instalando o ambiente e suportando bibliotecas. Para avaliar a eficácia dos modelos, o conjunto de dados é dividido em 70% para treinamento, 10% para validação e os 20% restantes utilizados para teste. As imagens de treinamento foram expandidas para 10.000 imagens de fundo de olho para cada tal.

**Resultados:**

Observou-se que o modelo ResNet50 alcançou acurácia de 97,1%, sensibilidade de 78,5%, especificidade de 98,5% e precisão de 79,7% e teve a melhor área sob a curva e pontuação final para classificar a categoria da catarata (área sob a curva=0,964, final=0,903). Em contraste, o modelo VGG16 alcançou uma precisão de 96,2%, sensibilidade de 56,9%, especificidade de 99,2% e precisão de 84,1%, área sob a curva 0,949 e pontuação final de 0,857.

**Conclusão:**

Esses resultados demonstram a capacidade das arquiteturas de rede neural convolucional pré-treinadas em identificar doenças oftalmológicas a partir de imagens de fundo de olho. ResNet50 pode ser uma boa solução para resolver problemas na detecção e classificação de doenças como glaucoma, catarata, hipertensão e miopia; Inceptionv3 para degeneração macular relacionada à idade e outras doenças; e VGG16 para retinopatia normal e diabética.

## INTRODUCTION

According to the World Report on Vision published by the World Health Organization, at least 2.2 billion people have vision impairment. The report emphasizes that at least one billion suffer from an impairment that could have been prevented or has yet to be addressed^(1)^.

Glaucoma is related to the degeneration of retinal ganglion cells and affects 7.7 million people^(2)^. It causes permanent blindness, and early detection is challenging. The number of people with glaucoma is projected to increase 1.3 times between 2020 (76 million) and 2030 (95.4 million), and those with age-related macular degeneration (AMD), 1.2 times between 2020 (195.6 million) and 2030 (243.3 million). Cataract, or the clouding of eye lens, affects approximately 94 million. Myopia, or nearsightedness, is a common cause of vision loss, and uncorrected myopia is the leading cause of distance vision impairment globally^(3)^. Other extreme vision impairments and blindness are usually generated by four ocular pathologies, namely, cataracts, diabetic retinopathy, AMD, and glaucoma^(4)^.

Ophthalmologists diagnose diseases based on pattern recognition using images of the fundus and its surrounding structures. This commitment of ophthalmology to disease detection using fundus images has laid the perfect groundwork for taking advantage of deep--learning architectures. Nowadays, attempts have been made to obtain clinical results using deep-learning architectures in the diagnosis, follow-up, and classification of common eye diseases. Recent studies have focused on deep-learning architectures on the classification of ophthalmological diseases such as diabetic retinopathy^(5,6)^, AMD^(7,8)^, glaucoma^(9,10)^, hypertension^(11,12)^, myopia^(13,14)^, and cataract^(15)^ through fundus imaging, visual field tests, or optical coherence tomography (OCT). Fundus screening allows for the detection of both ocular and systemic diseases, namely, diabetes, glaucoma, cataract, AMD, and other causes^(16)^.

In ophthalmology, enormous amounts of fundus images and patient-related data are available and produced daily. Among other eye diseases, cataract is one of the common causes of visual impairment and blindness worldwide, in which approximately 50% of cases have overall blindness. Therefore, early detection and prevention of cataracts can reduce visual impairment and blindness. In 2021, Khan et al. proposed an automated cataract detection system using the pretrained VGG model, and they achieved 97.47% accuracy in the test dataset^(17)^. The advancement of deep-learning in ophthalmology, such as in glaucoma, macular degeneration, diabetic retinopathy, corneal conditions, and age-related eye diseases, in addition to cataracts, has shown impressive results^(18-20)^. Influenced by these results, we set up three pretrained CNN architectures and determined which disease was successfully classified by which model.

Pretrained models were also known as transfer learning. Models are not created from scratch. Approximately one million images of ImageNet dataset are used in pretrained models. A pretrained model is just adapted to a new problem. Given the insufficient training and testing data for building a deep-learning model, a pretrained model is an option to automatically extract features. We chose VGG16, ResNet50, and Inceptionv3 for their high performance reported in the literature.

This study aimed to evaluate the classification performances of pretrained convolutional neural network (CNN) architectures using the grand challenge database called ocular disease intelligent recognition (ODIR).

## METHODS

**Figure 1:**
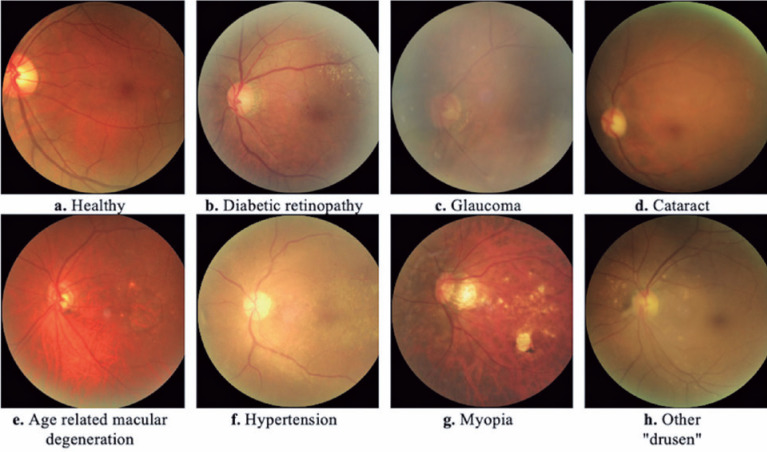
Fundus images with diagnostic keywords and associated ocular diseases.

The fundus images used in this study were obtained from ODIR sponsored by Peking University^(21)^. The publicly available dataset, containing “real” patient data from 487 hospitals, 26 cities in China, was collected by Shanggong Medical Technology Co., Ltd. The ODIR dataset contains fundus images of 5,000 left and right eyes of patients and ophthalmologists’ diagnostic keywords, namely, healthy, diabetic retinopathy, glaucoma, cataract, AMD, hypertensive retinopathy, myopia, and other diseases or anomalies. Fundus images with diagnostic keywords and associated ocular diseases are shown in Figure 1.

The dataset was divided into training, validation, and testing sets with 3,500, 500, and 1,000 pairs of fundus images, respectively. In the ODIR dataset, each patient’s left and right fundus images received ≥1 labels. The annotated classification labels of these patients were determined by the following rules. The classification labels of one patient depended on the left and right fundus images and corresponding diagnostic keywords. One patient was classified as normal if and only if both left and right diagnosis keywords were “normal fundus.” The classification labels were decided by the other fundus image when one of the fundus images was marked as “normal fundus.” All suspected diseases or abnormalities were labeled as other diseases. If two keywords “anterior segment image” and “no fundus image” were used, the image was not classified into any of the eight categories. The keywords “lens dust,” “optic disk photographically invisible,” “low image quality,” and “image offset” did not play a decisive role in determining the patient’s labels. The background of the left and right fundus images of patients with IDs [2174-2182] and [2957] was quite different from the others. Since these fundus images were preprocessed beforehand, they were not included in the model training. After identifying patients with these diagnostic keywords and preprocessed background images, 302 of 3,500 patients in the training set, 29 of 500 patients in the validation set, and 70 of 1,000 patients in the testing set were excluded. The class distribution of the eight categories of fundus images in the training, validation, and testing sets of the ODIR database is shown in Table 1.

**Table 1:** Class distribution of eight categories of fundus images in the training, validation, and testing sets of the ODIR database

Disease classification	Training (1/0)	Validation (1/0)	Test (1/0)	Total (1/0)
Healthy	1,001/2,197	147/324	274/656	1,422/3,177
Diabetic retinopathy	1,073/2,125	158/313	319/611	1,550/3,049
Glaucoma	197/3,001	27/444	51/879	275/4,324
Cataract	179/3,019	29/442	65/865	273/4,326
Age-related macular degeneration	163/3,035	25/446	48/882	236/4,363
Hypertension	103/3,095	16/455	30/900	149/4,450
Myopia	166/3,032	23/448	46/884	235/4,364
Others	883/2,315	131/340	263/667	1277/3,322
Total	3,198	471	930	4,599

1/0= disease positive/disease negative.

Following the left and right diagnostic keywords of the patients and the aforementioned rules, the patients were assigned to the disease positive or disease negative category for each class from eight different classes corresponding to the diagnostic keywords. Then, they were turned into binary classification problems, not multi-diagnostic problems. Accordingly, each patient’s fundus images were assigned to only one category in the training, validation, and testing sets, e.g., cataract or not cataract.

Gour et al. proposed two inputs and concatenated input CNN architecture for the multiclass multilabel fundus images of ophthalmologic diseases using transfer learning-based CNN approaches. They used four CNN architectures pretrained with two different optimizers and noted that the pretrained VGG16 architecture with the SGD optimizer performed better for multiclass multilabel fundus image classification in the ODIR database^(22)^. Wang et al proposed a multilabel classification ensemble model of fundus images based on CNN to directly detect one or more fundus diseases in fundus images. Each model consisted of two parts: a feature extraction network based on EfficientNet and a custom classification neural network for multilabel classification problems. Finally, the output probabilities of the different models were combined as the final recognition result. Experimental results showed that the model can be trained using fewer datasets, and good results can be obtained^(23)^.

Fundus images in the ODIR dataset were preprocessed before model training. A frame was created by determining the coordinates where the colored pixels were located. The parts with black pixels outside this frame were not included in the images. The left and right fundus image pixel widths of the patients were concatenated in the horizontal axis. The mirroring process was performed on the concatenated image. The height of the images combined on the horizontal axis was added to the height of the images obtained after mirroring on the vertical axis.

Table 1 shows that the frequency of glaucoma, cataract, AMD, hypertension, and myopia disease classes was very low. To eliminate the imbalance in class distribution, data augmentation was applied so that the fundus image frequencies of the eight disease classes were equal. First, from the Augmentor package functions in Python programming language, grid_width = 3, grid_height = 3, magnitude = 3, random distortion function with probability 0.25; secondly, grid_width = 3, grid_height = 3, magnitude = 3, corner = “bell”, method = “in” gaussian distortion function with the same probability; and finally skewed with probability 0.05; skew_tilt; skew_left_right; skew_top_bottom; skew_corner functions were used. In this process, we obtained 10,000 new fundus images in the newly created indices, with equal numbers in each subcategory of no disease “0” and disease present “1” in each disease category.

Input images obtained after image preprocessing and data augmentation were adapted to the dimensions used in the data input layers of the models, with 224 × 224 × 3 for VGG16 and ResNet50 and 299 × 299 × 3 for Inceptionv3. Newly created fundus images were used for each disease class in the input layers of these models. In these model architectures, the default learning rate was 0.001 for a batch size of 32, 0.9 for β_1_, 0.999 for β_2_, and 10^-8^ for ε, and an adaptive moment optimization algorithm was used.

To perform ophthalmological disease classification in the VGG16 model architecture, 1,000 classes in the fully connected layer in the ImageNet object classification were adapted to two classes. All layers except the last three fully connected layers of the architecture were frozen using pretrained layer weights. In the Inceptionv3 model architecture, the number of classes in the fully connected layer was adapted to two classes. All layers except the second Inception *C*, Inception*Aux*, Inception *D*, Inception *E* modules, and fully connected layer, which was the output, were frozen. In ResNet50, the weights up to the seventh layer of the architecture were frozen. These deep-learning architectures were trained for the classification of ophthalmological diseases by fine-tuning the ODIR fundus image set. Finally, the softmax layer was replaced with the customized layer for the ophthalmological disease classification with two classes.

The ODIR dataset had some limitations. As a fundus image dataset with real clinical applications, the use of 10,000 images could not adequately meet the need to develop a real-time deep-learning application. More fundus images are needed for a more accurate clinical diagnosis. This may enable our pretrained models to have better generalization ability. The rarity of some fundus diseases made it difficult to define these fundus diseases. In addition, although the ODIR dataset provided detailed diagnostic keywords for each fundus image, it was ultimately divided into eight categories. By making a more detailed subset classification of fundus diseases, a better understanding of the images belonging to the classifications can be achieved. In addition, the source of images was collected from a single ethnicity (Chinese only). Fundus data from different races or ethnicities are needed to have a better diversity of the dataset.

### Running environment

All deep-learning CNNs, pretrained on ImageNet, were trained and tested based on a publicly available CNN framework Pytorch. The coding and training steps were conducted in Google Colaboratory (Colab-Pro). This system is essential to this study because we can use NVIDIA Tesla-P100 GPU, with 25 gigabyte of random--access memory and 100 GB of memory in a cloud computing environment.

The following early stopping strategy was applied for all the classification experiments: the training procedure did not stop until the validation loss was continuously higher than the lowest validation loss for seven epochs.

### Evaluation metrics

Four evaluation metrics, including kappa, F1-score (F1), area under the curve (AUC), and their mean value, denoted as the final score, were used to evaluate the classification performance of the pretrained CNN models for ophthalmological diseases from fundus images. The kappa coefficient was used for consistency check, and it ranged from -1 to 1. F1 is the harmonic mean of precision and recall. Since kappa and F1 only consider a single threshold, the output of the classification networks is probabilistic; thus, we used the area under the receiver operating characteristics curve. All these four metrics were calculated by the sklearn package.

## RESULTS

This study focused on the classification performance of pretrained VGG16, Inceptionv3, and ResNet50 models. After training these models, the weights were saved for the prediction of the previously unseen dataset of test images. The trained model was then used for the classification of the test images as ophthalmological disease labels based on model accuracy and loss. Table 2 describes the accuracy and loss achieved for the training and validation sets in the last epoch when the pretrained models were trained.

**Table 2:** Training and validation performances of the pretrained models in the last epoch for each ophthalmological disease classification

		Training		Validation	
Model	Disease classification	Acc	Loss	Acc	Loss
	Healthy	0.719	0.476	0.597	1.193
	Diabetic retinopathy	0.735	0.461	0.684	0.999
	Glaucoma	0.954	0.171	0.892	0.845
**VGG16**	Cataract	0.981	0.154	0.968	0.129
	Age-related macular degeneration	0.935	0.225	0.945	0.548
	Hypertension	0.946	0.203	0.955	0.461
	Myopia	0.995	0.273	0.972	3.324
	Others	0.646	0.589	0.556	0.991
	Healthy	0.997	0.016	0.660	2.263
	Diabetic retinopathy	0.994	0.024	0.720	1.721
	Glaucoma	0.997	0.013	0.915	0.863
**Inceptionv3**	Cataract	0.998	0.006	0.968	0.237
	Age-related macular degeneration	0.999	0.004	0.962	0.597
	Hypertension	0.996	0.020	0.955	0.798
	Myopia	0.999	0.005	0.972	0.289
	Others	0.992	0.039	0.667	2.455
	Healthy	0.991	0.028	0.648	1.890
	Diabetic retinopathy	0.992	0.022	0.701	2.263
	Glaucoma	0.999	0.004	0.943	0.876
**ResNet50**	Cataract	0.999	0.001	0.981	0.428
	Age-related macular degeneration	0.998	0.006	0.960	0.448
	Hypertension	0.998	0.007	0.960	0.859
	Myopia	0.999	0.002	0.979	0.254
	Others	0.995	0.016	0.709	2.423

Acc= accuracy.

Table 3 displays the confusion matrix of the pretrained CNN models with a softmax classifier. Each row of the confusion matrix represents instances in an actual/true class, and each column of the matrix represents instances in a predicted class. The values in the main diagonal of the matrix represent instances where the model can accurately predict the class to which an image belongs (true negative [TN] and true positive [TP]). On the contrary, all values in the confusion matrix, except the major diagonal, represent cases where the model misclassifies an image (false negative [FN] and false positive [FP]).

**Table 3:** Confusion matrix of pretrained convolutional neural network architectures for ophthalmological diseases.

Disease classification		Validation data						Test data					
		VGG16		Inceptionv3		ResNet50		VGG16		Inceptionv3		ResNet50	
		N	P	N	P	N	P	N	P	N	P	N	P
Healthy	**N**	199(TN)	125(FP)	174(TN)	150(FP)	255(TN)	69(FP)	443(TN)	213(FP)	431(TN)	225(FP)	519(TN)	137(FP)
	**P**	70(FN)	77(TP)	39(FN)	108(TP)	87(FN)	60(TP)	97(FN)	177(TP)	102(FN)	172(TP)	160(FN)	114(TP)
Diabetic retinopathy	**N**	236(TN)	77(FP)	279(TN)	34(FP)	301(TN)	12(FP)	428(TN)	183(FP)	585(TN)	26(FP)	580(TN)	31(FP)
	**P**	81(FN)	77(TP)	95(FN)	63(TP)	129(FN)	29(TP)	140(FN)	179(TP)	266(FN)	53(TP)	260(FN)	59(TP)
Glaucoma	**N**	410(TN)	34(FP)	382(TN)	62(FP)	430(TN)	14(FP)	842(TN)	37(FP)	855(TN)	24(FP)	854(TN)	25(FP)
	**P**	23(FN)	4(TP)	17(FN)	10(TP)	21(FN)	6(TP)	38(FN)	13(TP)	38(FN)	13(TP)	36(FN)	15(TP)
Cataract	**N**	435(TN)	7(FP)	437(TN)	5(FP)	438(TN)	4(FP)	858(TN)	7(FP)	853(TN)	12(FP)	852(TN)	13(FP)
	**P**	6(FN)	23(TP)	3(FN)	26(TP)	5(FN)	24(TP)	28(FN)	37(TP)	18(FN)	47(TP)	14(FN)	51(TP)
Age-related macular degeneration	**N**	440(TN)	6(FP)	432(TN)	14(FP)	424(TN)	22(FP)	857(TN)	25(FP)	860(TN)	22(FP)	819(TN)	63(FP)
	**P**	22(FN)	3(TP)	12(FN)	13(TP)	14(FN)	11(TP)	41(FN)	7(TP)	34(FN)	14(TP)	26(FN)	22(TP)
Hypertension	**N**	452(TN)	3(FP)	455(TN)	0(FP)	440(TN)	15(FP)	898(TN)	2(FP)	900(TN)	0(FP)	840(TN)	60(FP)
	**P**	16(FN)	0(TP)	16(FN)	0(TP)	14(FN)	2(TP)	30(FN)	0(TP)	30(FN)	0(TP)	18(FN)	12(TP)
Myopia	**N**	444(TN)	4(FP)	443(TN)	5(FP)	439(TN)	9(FP)	858(TN)	26(FP)	849(TN)	35(FP)	858(TN)	26(FP)
	**P**	9(FN)	14(TP)	4(FN)	19(TP)	5(FN)	18(TP)	21(FN)	25(TP)	12(FN)	34(TP)	14(FN)	32(TP)
Others	**N**	208(TN)	132(FP)	257(TN)	83(FP)	277(TN)	63(FP)	423(TN)	244(FP)	636(TN)	31(FP)	595(TN)	72(FP)
	**P**	81(FN)	50(TP)	88(FN)	43(TP)	90(FN)	41(TP)	129(FN)	134(TP)	230(FN)	33(TP)	213(FN)	50(TP)

N= negative; P= positive; TN= true negative; FN= false negative; TP= true positive; FP= false positive.

As shown in Table 3, the pretrained ResNet50 model had the highest correct classification (TN + TP) and the lowest misclassification (FN + FP) for healthy, other, and glaucoma disease classes in the validation data. In addition, the pretrained ResNet50 model had the highest correct classification and the lowest misclassification for diabetic retinopathy, cataract, and myopia disease classes in addition to healthy and glaucoma disease classes in the testing data. The pretrained Inceptionv3 model had the highest correct classification and the lowest misclassification for diabetic retinopathy, cataract, AMD, hypertension, and myopia disease classes in the validation data. In addition, the pretrained Inceptionv3 model had the highest correct classification and the lowest misclassification for AMD, hypertension, and other disease classes in the test data.

The standard measures of performance accuracy, precision, sensitivity, and specificity are calculated from the confusion matrix, and four evaluation metrics including kappa, F1, AUC, and their mean value (final score) are presented in the validation and testing sets in Tables 4 and 5, respectively.

**Table 4:** Evaluation of classification performances of pretrained convolutional neural network architectures for ophthalmological diseases in the validation set

		Ophthalmological disease classification							
**Model**	**Metrics**	N	D	G	C	A	H	M	O
	Acc	0.586	0.665	0.879	0.972	0.941	0.960	0.972	0.548
	Prec	0.381	0.500	0.105	0.767	0.333	0.000	0.778	0.275
	Sens	0.524	0.487	0.148	0.793	0.120	0.000	0.609	0.382
**VGG16**	Spec	0.614	0.754	0.923	0.984	0.987	0.993	0.991	0.612
	Kappa	0.125	0.243	0.060	0.765	0.153	-0.011	0.669	-0.006
	F1	0.586	0.665	0.879	0.972	0.941	0.960	0.972	0.548
	AUC	0.617	0.709	0.719	0.988	0.720	0.604	0.967	0.504
	Final	0.443	0.539	0.553	0.908	0.605	0.518	0.870	0.349
	Acc	0.599	0.726	0.832	0.983	0.945	0.966	0.981	0.637
	Prec	0.419	0.649	0.139	0.839	0.481	0.000	0.792	0.341
	Sens	0.735	0.399	0.370	0.897	0.520	0.000	0.826	0.328
**Inceptionv3**	Spec	0.537	0.891	0.860	0.989	0.969	1.000	0.989	0.756
	Kappa	0.225	0.321	0.129	0.858	0.471	0.000	0.798	0.085
	F1	0.599	0.726	0.832	0.983	0.945	0.966	0.981	0.637
	AUC	0.691	0.758	0.662	0.987	0.796	0.690	0.991	0.556
	Final	0.505	**0.602**	0.541	**0.943**	**0.737**	0.552	**0.923**	0.426
	Acc	0.669	0.701	0.926	0.981	0.924	0.938	0.970	0.675
	Prec	0.465	0.707	0.300	0.857	0.333	0.118	0.667	0.394
	Sens	0.408	0.184	0.222	0.828	0.440	0.125	0.783	0.313
**ResNet50**	Spec	0.787	0.962	0.968	0.991	0.951	0.967	0.980	0.815
	Kappa	0.202	0.178	0.217	0.832	0.339	0.089	0.704	0.136
	F1	0.669	0.701	0.926	0.981	0.924	0.938	0.970	0.675
	AUC	0.682	0.695	0.678	0.988	0.815	0.745	0.989	0.608
	Final	**0.518**	0.525	**0.607**	0.933	0.693	**0.591**	0.888	**0.473**

A: Age= age-related macular degeneration; Acc= accuracy; AUC= area under the curve; C= cataract; D= diabetic retinopathy; Final= mean values of F1, AUC, and kappa; G= glaucoma; H= hypertension; M= myopia; N, healthy; O= others; Prec= precision; Sens= sensitivity; Spec= specificity.

**Table 5:** Evaluation of classification performances of pretrained convolutional neural network architectures for ophthalmological diseases in the testing set

		Ophthalmological disease classification							
**Model**	**Metrics**	N	D	G	C	A	H	M	O
	Acc	0.667	0.653	0.919	0.962	0.929	0.966	0.949	0.599
	Prec	0.454	0.494	0.260	0.841	0.219	0.000	0.490	0.354
	Sens	0.646	0.561	0.255	0.569	0.146	0.000	0.543	0.510
**VGG16**	Spec	0.675	0.700	0.958	0.992	0.972	0.998	0.971	0.634
	Kappa	0.286	0.253	0.215	0.660	0.139	-0.004	0.489	0.127
	F1	0.667	0.653	0.919	0.962	0.929	0.966	0.949	0.599
	AUC	0.717	0.677	0.726	0.949	0.578	0.654	0.861	0.586
	Final	**0.557**	**0.528**	0.620	0.857	0.549	0.538	0.767	0.437
	Acc	0.648	0.686	0.933	0.968	0.940	0.968	0.949	0.719
	Prec	0.433	0.671	0.351	0.797	0.389	0.000	0.493	0.516
	Sens	0.628	0.166	0.255	0.723	0.292	0.000	0.739	0.125
**Inceptionv3**	Spec	0.657	0.957	0.973	0.986	0.975	1.000	0.960	0.954
	Kappa	0.252	0.151	0.261	0.741	0.302	0.000	0.566	0.102
	F1	0.648	0.686	0.933	0.968	0.940	0.968	0.949	0.719
	AUC	0.711	0.726	0.685	0.948	0.748	0.706	0.940	0.621
	Final	0.537	0.521	0.626	0.885	**0.663**	0.558	0.818	**0.481**
	Acc	0.681	0.687	0.934	0.971	0.904	0.916	0.957	0.694
	Prec	0.454	0.656	0.375	0.797	0.259	0.167	0.552	0.410
	Sens	0.416	0.185	0.294	0.785	0.458	0.400	0.696	0.190
**ResNet50**	Spec	0.791	0.949	0.972	0.985	0.929	0.933	0.971	0.892
	Kappa	0.212	0.162	0.296	0.775	0.284	0.199	0.593	0.098
	F1	0.681	0.687	0.934	0.971	0.904	0.916	0.957	0.694
	AUC	0.703	0.643	0.762	0.964	0.702	0.764	0.959	0.575
	Final	0.532	0.497	**0.664**	**0.903**	0.630	**0.626**	**0.836**	0.456

A= age-related macular degeneration; Acc= accuracy; AUC= area under the curve; C= cataract; D= diabetic retinopathy; Final= mean values of F1, AUC, and kappa; G= glaucoma; H= hypertension; M= myopia; N= healthy; O= others; Prec, precision; Sens= sensitivity; Spec= specificity.

Table 4 shows that the pretrained Inceptionv3 model for the validation set achieved an accuracy of 98.3%, sensitivity of 89.7%, specificity of 98.9%, and precision of 83.9% and had the best final score to classify cataract (AUC=0.987, final=0.943). To classify myopia, this model achieved an accuracy of 98.1%, sensitivity of 82.6%, specificity of 98.9%, and precision of 79.2% and had the best final score (AUC=0.991, final=0.923). To classify AMD, this model achieved an accuracy of 94.5%, sensitivity of 52%, specificity of 96.9%, and precision of 48.1% and had the best final score (AUC=0.796, final=0.737). To classify diabetic retinopathy, this model achieved an accuracy of 72.6%, sensitivity of 39.9%, specificity of 89.1%, and precision of 64.9% and had the best final score (AUC=0.758, final=0.602). The pretrained VGG16 model did not reach the best final score for any classification category in the validation set.

Table 5 shows the classification performance of the pretrained CNN architectures for ophthalmological diseases in the testing set. Moreover, the classification performances of normal and diabetic retinopathy reached the highest final score with the pretrained VGG16 model compared with ResNet50 and Inceptionv3 models (AUC_N_=0.717, Final_N_=0.557; AUC_D_=0.677, Final_D_=0.528). The pretrained Inceptionv3 model achieved the highest final score in classifying AMD and other diseases compared with VGG16 and ResNet50 (AUC_A_=0.748, Final_A_=0.663; AUC_O_=0.621, Final_O_=0.481). The pretrained ResNet50 model achieved the highest final score in classifying glaucoma, cataract, hypertension, and myopia compared with VGG16 and Inceptionv3 (AUC_G_=0.762, Final_G_=0.664; AUC_C_=0.964, Final_C_=0.903; AUC_H_=0.764, Final_H_=0.626, AUC_M_=0.959, Final_M_=0.836).

Cataract and myopia classes achieved the highest AUC and final scores when the performance results of the models in the validation and testing sets were evaluated in general (Tables 4 and 5). Although the accuracy of the models corresponding to glaucoma, AMD, and hypertension classifications was >0.80, the low precision, sensitivity, and specificity values of these disease classifications affected the AUC and thus the final score. The F1 score value was >0.90 in glaucoma, cataract, AMD, hypertension, and myopia classes with data imbalances. For the normal category, if both fundus images were labeled normal, this classification had an issue because the patient was included in the normal category, which affected the results. In addition, patients were labeled with a total of 117 diagnostic keywords, including eight disease classes. Therefore, the performance of the pretrained model for the other diseases category was quite low. To improve the performance of the models in this category, a new classification category suitable for each diagnostic keyword is needed, more fundus images containing these diagnostic keywords should be collected.

## DISCUSSION

Many datasets consist of high-quality images that were captured under controlled, non-standard conditions. Arguably, algorithms trained on such datasets will perform poorly because the images may not be directly comparable and environmental and hardware details may differ. On the contrary, the ODIR dataset addresses these issues, “real-life” patient data were collected from different hospitals/medical centers, and images were captured using different camera models under various nontypical conditions. As a result, the noise caused by those variations makes it very difficult for the algorithms to conduct an accurate and effective analysis.

The development of large training and validation datasets is one of the many necessary steps toward the development of robust and accurate artificial intelligence (AI) models. However, most of the datasets lack sufficient data or suffer from the imbalance between their classes. The ODIR dataset has data imbalance in disease classes of glaucoma, cataract, AMD, hypertension, and myopia. Thus, several ways can be employed to overcome this issue, such as using augmentation techniques and leveraging the knowledge of pretrained models on large datasets, such as ImageNet^(24,25)^. In this study, we used both pretrained models with weights obtained in ImageNet and appropriate data augmentation techniques.

AI, especially deep-learning-based methods, holds promise for improving and accelerating advances in heal-thcare. However, several important constraints should be addressed to facilitate its adoption in clinical settings^(26)^. Apart from the traditional methods to evaluate model performance, i.e., accuracy metrics, several others have been proposed as important for the acceptance of AI models^(27)^. The transition from traditional machine-learning approaches to deep-learning models has improved the performance of such analyses^(28)^.

**Figure 2:**
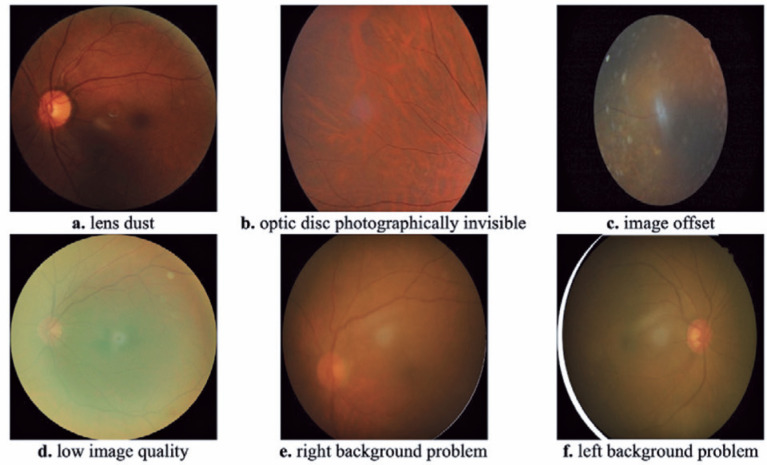
Examples of fundus images not included in the model training because of the lens dust, photographically invisible optic disk, image offset, low image quality, and right and left background problems.

The classification performance results of deep-learning models can be affected by dataset quality, labeling process, dataset heterogeneity, and dataset class imbalance^(29)^. The ODIR dataset was constructed by collecting images from different hospitals and clinics in China. Fundus images were captured by various cameras, such as Canon, Zeiss, and Kowa, which varied the resolutions of images. Ensuring that the quality of the captured fundus image is similar to the actual fundus is challenging. Fundus cameras may fail to capture important features responsible for disease identification, images had diffe-rent resolutions and angles, and fundus images had heterogeneous sizes. The models trained on such data may fail in categorizing images belonging to the same class. This dataset also contained out-of-focus blurred images and had artifacts that interfere with the training images, as shown in Figure 2. We detected these images using the keywords given in the data preprocessing step and did not include them in model training, as they did not contribute or play a decisive role in determining a patient’s disease. In addition, we created a frame by determining the coordinates where the colored pixels are located. We did not include the black pixel portions outside this frame and tried to make all image sizes homogeneous.

Instead of training the network from scratch, we used pretrained models trained on ImageNet and fine-tuned them on the fundus image data. We used pretrained models as feature extractors, based on the assumption that the primary layers of the models provide relevant baseline features. Another alternative approach was to eliminate the imbalance in class distribution by generating synthetic fundus image data equally in each classification category based on the augmentation strategy specified in the Methods.

To our knowledge, this is the first study that evalua-ted model performances using pretrained models for two-class classification in each disease category of eight ophthalmological diseases after obtaining a total of 10,000 fundus images per class using data augmentation techniques in the ODIR dataset. According to the results, CNN training in ophthalmology may be a viable choice because publicly available datasets are increasing.

In this study, we evaluated the performance of pretrained CNN architectures of VGG16, Inceptionv3, and ResNet50 for the automated classification of clinical fundus images in the newly publicly available ODIR dataset with multi-disease annotations. As shown in the experiments, ResNet50 and Inceptionv3 provided higher final scores than VGG16. ResNet50 requires fewer parameters and time to obtain classification results. ResNet50 can be a good architecture to solve problems in disease detection and classification of glaucoma, cataract, hypertension, and myopia; Inceptionv3 for AMD and other diseases; and VGG16 for normal and diabetic retinopathy. These deep-learning architectures might be efficient solutions for the optimization and classification of diseases using fundus images in real-life clinical settings.
